# Mechanical Behavior of Thermoplastic Starch: Rationale for the Temperature-Relative Humidity Equivalence

**DOI:** 10.3390/polym14132531

**Published:** 2022-06-21

**Authors:** Lise Leroy, Gregory Stoclet, Jean-Marc Lefebvre, Valerie Gaucher

**Affiliations:** CNRS, INRAE, Centrale Lille, UMR 8207-UMET-Unité Matériaux et Transformations, University of Lille, F-59000 Lille, France; lise.leroy@macopharma.com (L.L.); jean-marc.lefebvre2@univ-lille.fr (J.-M.L.)

**Keywords:** thermoplastic starch, mechanical behavior, humidity

## Abstract

This paper aimed at understanding and rationalizing the influence of both temperature and relative humidity on the mechanical behavior of thermoplastic starch (TPS). DMA experiments revealed that water molecules impact the crosslinking network by reducing the intermolecular hydrogen bond density, resulting in a less dense entanglement network. In addition, the in-situ X-ray characterization during hydration of starch revealed structural changes, which were ascribed to conformational changes in the starch chain, due to their interaction with the uptake water molecules. Finally, the study of TPS uniaxially stretched at different temperatures and humidity showed that the mechanical behavior of TPS could be rationalized by considering the ΔT parameter, which corresponds to the temperature difference between the drawing temperature and the glass transition temperature of TPS.

## 1. Introduction

Starch is a cheap and abundant natural polymer. This polysaccharide can be transformed from its native granule state into thermoplastic starch (TPS) using conventional methods in the field of polymer processing, such as extrusion [1,2,3,4,5]. Nevertheless, the use of plasticizers is required in order to allow melt processing of starch below the degradation temperature. Due to the highly hydrophilic character of starch, water is the natural plasticizer of starch. Other plasticizers, such as polyols (glycerol, sorbitol, mannitol and so on) or, more recently, ionic liquids are also widely used [6,7]. In addition to the processing issues, plasticizers also impact the solid-state behavior of TPS.

Thermoplastic starch may provide a reliable alternative for substitution of commodity plastics in a rather broad range of applications, thus, justifying the large number of studies devoted to the characterization of this agro-sourced material. The mechanical behavior of TPS has already been the subject of numerous works. Nevertheless, an overview of the literature reveals that most studies deal with the mechanical behavior only at room temperature, the evolution of the properties being tuned by varying the plasticizer content [1,8]. A general trend is that the increase in plasticizer content results in an improvement of ductility, combining an increase of the deformation at break and a decrease of strength. High plasticizer contents are generally required for sufficiently decreasing T_g_ so as to enable the occurrence of ductile behavior at room temperature. However, it is not usually possible to continuously decrease T_g_ by increasing plasticizer content. Indeed, for high plasticizer amounts, the occurrence of some phase separation phenomenon impacts the mechanical behavior [9,10]. Mikus et al. have shown that plastic deformation mechanisms of plasticized wheat starch materials involve various volumetric strains which could be attributed to an anti-plasticization effect at small plasticizer content, to a phase separation phenomenon, in the case of excess of plasticizer and/or to retrogradation of starch [11]. 

The influence of the relative humidity, under which TPS is stored, has also been investigated [12,13]. In the case of glycerol plasticized waxy maize thermoplastic starch, Van Soest et al. reported a linear decrease of the Young’s modulus E for water contents up to 20 wt%. Regarding the deformation at break, they observed a brittle behavior for water contents below 10 wt% and then a linear increase with between 10 and 20 wt% of water content. The high sensitivity of TPS to the strain rate was also pointed out in this work. The latter is explained by structural considerations regarding the ability of the polymer chain segments to slip past each other depending on the kinetics of locking/unlocking of local intermolecular interactions, such as H-bonds. 

The botanical origin of starch directly governs its composition and, more specifically, the amylose/amylopectine ratio. Hulleman et al. reported an increase in the elongation at break with amylopectine content [14]. By contrast, Lourdin et al. linked the latter phenomenon to amylose content. They attributed this result to the linear conformation of amylose that favors the formation of entanglements [15]. 

As previously pointed out, a main feature of the aforementioned studies is that they generally investigated the mechanical behavior of TPS at room temperature, as a function of plasticizer content. In other words, probing the influence of draw temperature on the mechanical behavior of TPS at constant plasticizer content remains a poorly addressed topic. A study by Dieulot et al. highlighted the lack of available data in a broad range of stretching conditions [16]. In their work on the classification and prediction of mechanical behavior of starch-based films, the authors gathered 322 observations from literature as a function of starch origin, aging conditions or plasticizer content but not as a function of temperature. Studies dealing with the shape memory behavior of starch from Véchambre et al. showed that deformation of water and/or glycerol plasticized potato starch at temperatures varying from 50 °C to 115 °C led to an orientation of the macromolecular chains towards stretching direction [17,18]. Nevertheless, the mechanical behavior during drawing at these temperatures was not depicted and discussed. To our knowledge, only a work from Coativy et al. has reported the mechanical behavior of water plasticized potato starch and its nanocomposites when drawn at 90 °C, i.e., at T_g_ + 20 °C [19]. Under these conditions, TPS behaves as a rubbery material and is characterized by a relatively high strain at break (i.e., 150%) and low strength. 

In this context, the present paper is focused on the influence of stretching conditions, i.e., both temperature and relative humidity, on the drawing behavior of water plasticized amorphous TPS. 

## 2. Materials and Methods

### 2.1. Extrusion of Potato Starch

Thermoplastic potato starch was obtained by extrusion of the native potato starch supplied by Roquette Frères (Lestrem, France). The amylose/amylopectine weight ratio was 23/77. Prior to extrusion, the potato native powder was hydrated to adjust the moisture content at 35 wt% d.b. (dry basis) as determined by TGA analyses. For this purpose, the appropriate quantity of distilled water was added and blended to the as-received powder. The hydrated powder was then stored for 2 days at 5 °C in order to reach equilibrium. Extrusion was performed using a SCAMIA single-screw device (Rheoscam Type 20.11d, Crosne, France). The die temperature was set at 110 °C; the screw speed was set at 25 rpm and the specific mechanical energy, measured from the torque of the shaft, was of the order of 170 kJ/kg. Extruded rods were then kept in a chamber containing a saturated sodium bromide solution (NaBr, R.H. 58% at 20 °C) for one week at least in order to stabilize the water content and to prevent any retrogradation phenomenon. 

### 2.2. Elaboration of TPS Samples and Conditioning

All characterizations were performed on thermo-compressed samples. This step required a rehydration of the stabilized extruded rods by storing them overnight at 89% R.H. and 20 °C. The hydrated rods were then compression molded at 120 °C under 180 bar to obtain 1 mm thick sheets using a Darragon hot-press. Compression-molded samples were then stored at 20 °C under two relative air humidity conditions (58 and 89% RH) for at least 3 days before analyses in order to reach the equilibrium state (duration determined from the water absorption kinetics obtained from Dynamic Vapor sorption experiments). Under these conditions, a TPS sample stored at 20 °C under 58% RH reached a water content of 14 wt% h.b. (humid basis), while a TPS sample stored at 20 °C under 89% RH had a water content of 18 wt% h.b. (values determined by TGA experiments). Moreover, it was confirmed by means of WAXS experiments that no structural evolutions were induced during storage. For the sake of clarity, thermoplastic potato starch samples stored at 20 °C and relative humidity of 58 and 89% RH were denoted TPS58 and TPS89, respectively.

### 2.3. Thermal Behaviour

Thermal properties were evaluated by means of both Differential Scanning Calorimetry (DSC) experiments using a DSC7 (Perkin Elmer, Waltham, MA, USA) apparatus and Dynamic Mechanical Analysis (DMA) in oscillatory tensile mode using a RSA3 (TA Instruments, New Castle, DE, USA) apparatus. DSC experiments were performed in the 25 to 180 °C temperature range at a heating rate of 10 °C/min. Temperature and heat flow were calibrated with a high purity indium sample using standard procedures. Experiments were carried out under nitrogen flow on about 10 mg samples placed in sealed steel pans. DMA experiments were carried out in the −150 to 120 °C temperature range at a heating rate of 2 °C/min and a frequency of 1 Hz on 1 mm thick parallelepiped specimens (13 × 38 mm²). In order to keep the water amount constant inside the samples, the latter were covered with a silicone-based hydrophobic grease to prevent moisture departure during experiments.

### 2.4. Structural Analysis

Structural characterization was performed using Wide Angle X-ray Scattering (WAXS) at room temperature in transmission mode. The CuK_α_ radiation (λ = 1.54 Å) was generated by a Genix micro-source (Xenocs, Grenoble France) and collimated and monochromatized by a FOX2D-12Inf optic (Xenocs, Grenoble; France). WAXS patterns were recorded on a CCD detector (Photonic Sciences, Saint Leonards-on-sea, UK). Standard corrections were applied to the patterns before their treatment. The intensity profiles were obtained by 360° azimuthal integration of the 2D patterns using the fit2D software^®^ (software version number: 16.041, creator A P Hammersley, Grenoble, France). 

### 2.5. Uniaxial Tensile Behavior

Uniaxial tensile testing was performed as a function of both temperature and relative humidity on dumbbell shaped specimens (20 mm long and 5 mm width) cut off from the thermo-compressed sheets. An INSTRON 5966 apparatus equipped with a temperature- and humidity-controlled oven (SERVATIN, Balaruc-les-Bains, France) was used. Tensile tests were carried out at an initial strain rate of 1.10^−2^ s^−1^ between 25 and 90 °C with a relative humidity varying from 30 to 90% RH. Prior to drawing, TPS89 samples were held in the oven for 10 min in order to reach an equilibrium state. To take into account the evolution of the water content of the samples during the stabilization step, the sample weight before and after stabilization in the climatic oven was determined using a precision scale. The corresponding glass transition temperature values were then deduced from the results reported by Bizot et al. [20], which depicted the evolution of T_g_ as a function of water content. For each drawing condition, experiments were reproduced at least 3 times in order to ensure reproducibility. 

## 3. Results and Discussion

### 3.1. Characterization of TPS58 and TPS89

**Figure 1:**
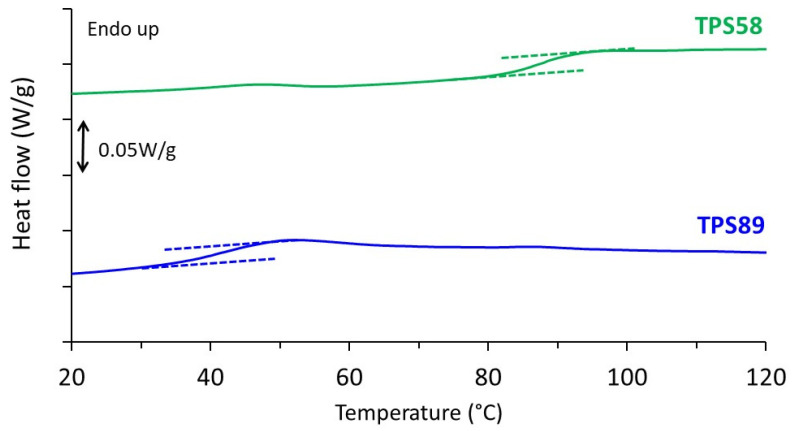
DSC thermograms of TPS stored at 20 °C and relative humidity of 58 and 89% RH recorded during heating at 10 °C/min.

Thermal behavior of TPS58 and TPS89 is reported in Figure 1. Only one heat capacity jump was evidenced in both thermograms. As expected, the glass transition temperature (T_g_) was lower for the sample stored at the higher relative humidity, showing the plasticizing effect of the water molecules. The T_g_ values were 95 and 45 °C for samples stored at 58% RH and 89% RH, respectively, and were in good agreement with the ones previously reported in literature [20,21]. Both values were above ambient temperature which may explain why no major structural evolution occurred during storage. No melting peak was observed up to a temperature of 200 °C, indicative of fully amorphous samples in both cases. This result was consistent with WAXS analyses, as will be shown later. A slight endothermic peak was observed around 45 °C in the case of TPS58. This phenomenon had already been reported by other authors and is attributed to a physical aging phenomenon [22]. 

**Figure 2:**
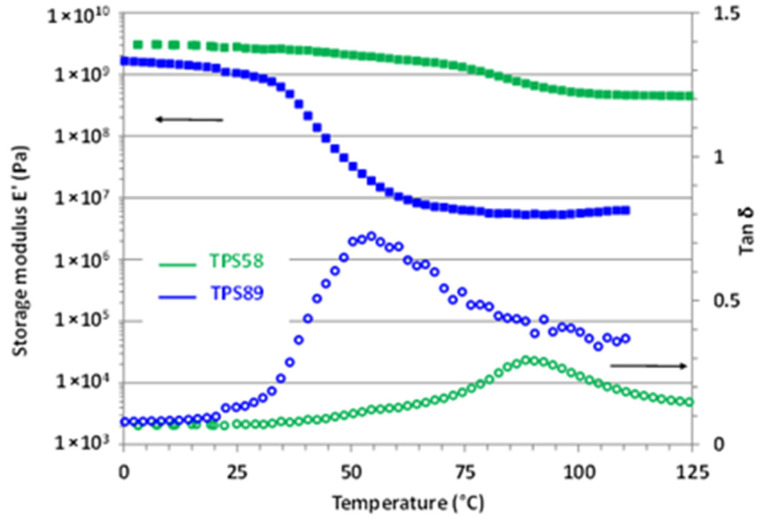
Evolution of the storage modulus E’ and loss factor tanδ as a function of temperature for TPS58 and TPS89.

The influence of water content on the viscoelastic properties of TPS was also investigated. The evolution of both storage modulus E’ and loss factor tanδ for TPS58 and TPS89 samples are depicted in Figure 2.

At low temperature, both TPS58 and TPS89 exhibited a storage modulus around 3 and 2 GPa, characteristic of a glassy material. The large drop of E’, associated with the occurrence of the main α relaxation, was observed around 40 °C for TPS89 and around 90 °C for TPS58 in agreement with previous DSC results. Worth noting is that the amplitude of the E’ decrease strongly varied depending on the water content of TPS. While E’ in the rubbery plateau was equal to about 50 MPa for TPS89 (18 wt% water content), the corresponding value for TPS58 (14 wt% water content) was in the range of 500 MPa, a value rather characteristic of a semi-crystalline or crosslinked polymer.

For both TPS58 and TPS89, the rubber modulus was larger than the ones generally reported in the case of amorphous thermoplastic polymers (in the fraction of MPa range) [23]. Moreover, plasticization of an amorphous polymer involves a decrease in T_α_ but, generally, does not significantly infer on the level of the storage modulus in the rubbery plateau [24,25]. 

According to the theory of rubber elasticity, the value of the storage modulus in the rubbery plateau region is linked to the entanglement molecular weight, *M_e_*, by the following equation:

(1) $$M_{e}\propto \;\frac{\rho RT}{G_{N}^{0}}$$

where ρ is the specific gravity of the material, *R* the perfect gas constant, *T* the temperature and $G_{N}^{0}$ the rubber modulus value taken at minimum tan δ [26]. 

Relying on the above equation, results showed that *M_e_* for TPS58 was 10 times lower than that of TPS89. In other words, the macromolecular network of TPS58 had a much smaller mesh size than the one of TPS89. Basically, for a fully linear amorphous polymer, *M_e_* is related to the physical links originating from the macromolecular entanglements. In fact, in the case of amorphous TPS, the effective network comprises not only the entanglement network of the linear amylose component, but also the contribution of both amylose and amylopectine intermolecular interactions through H-bonds. It is worth noting that the TPS58 and TPS89 samples originated from the same batch, and therefore the only difference lay in their water content. The results, thus, evidenced that water molecules impact the crosslinking network. Considering the high level of H-bond interactions in the starch macromolecules, we might thus postulate that water molecules reduced intermolecular hydrogen bond density, resulting in a higher effective *M_e_* at increasing water content, in agreement with the fact that TPS89 displayed the lowest rubber modulus.

In summary, these results clearly showed that the water molecules not only acted as classical plasticizers, by decreasing the T_g_ value, but also strongly inferred on the macromolecular network by decreasing H-bond density. We might, thus, expect that the water content would also have an impact on the mechanical behavior of TPS when drawn in the rubbery state. 

**Figure 3:**
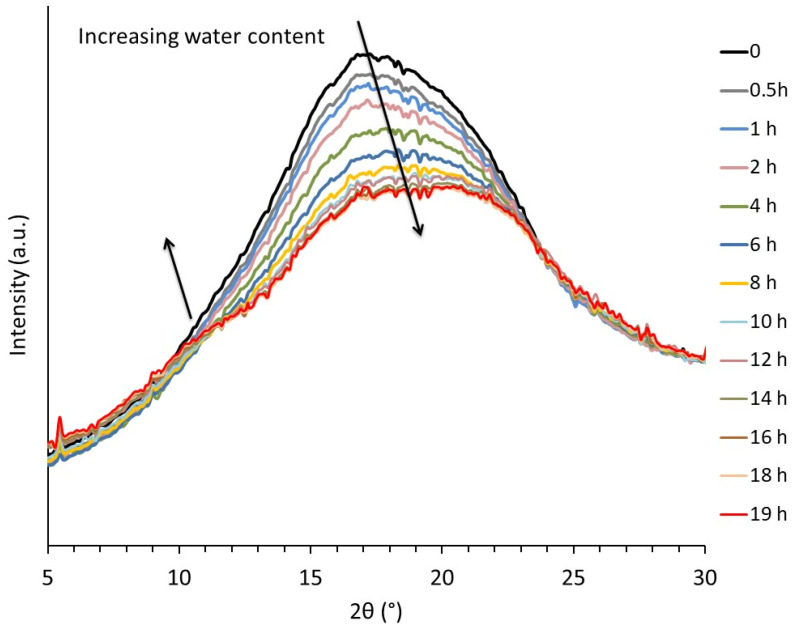
Evolution of the integrated intensity profile during re-hydration of a dry TPS samples stored at 89% RH, T = 20 °C.

Some clues concerning the structural modifications induced by the presence of water were provided by WAXS analysis. Figure 3 depicts the real-time evolution of the integrated intensity profiles of an initially dry TPS sample as a function of storage time at 20 °C under 89% RH. During storage, the water content increased from ≈0 to 18 wt% after 19 h. This was assessed by measuring the evolution of the sample weight as a function of storage time. It clearly appeared that water absorption influenced the structural organization of the macromolecules in the amorphous phase. A marked decrease in peak intensity was observed in the 15–20° 2θ range in parallel to the emergence of a peak shoulder in the 10–12° 2θ range during the first 12 h of storage. Consistent results were reported in the case of the structural evolution of TPS upon drying by Bayer et al. [27]. For further insights into their findings, these authors proposed a peak profile analysis thanks to which they linked the evolution of the integrated intensity profiles to structural aspects. In their analysis, they decomposed the amorphous starch scattering into three components: two Gaussian curves having a maximum located at around 11–12° and 19–20°, associated with disordered starch single helices, and a third peak having a maximum at approximatively 15–16°, corresponding to the scattering of disordered regions containing double helices. On this basis, they suggested the co-existence of two kinds of crosslink/entanglement points, the latter resulting in the classical macromolecular entanglement network; the former originating from the formation of double helices physical crosslinks, inducing network densification. Based on Bayer et al.’s analysis, one may conclude that penetration of water molecules into amorphous phase leads to an increase in the single helices content at the expense of the double helices. To sum up, assuming that double helices act as crosslink points, the reduction of their number will induce a decrease of the rubbery modulus, in agreement with the observed viscoelastic behavior. 

### 3.2. Mechanical Behaviour 

**Figure 4:**
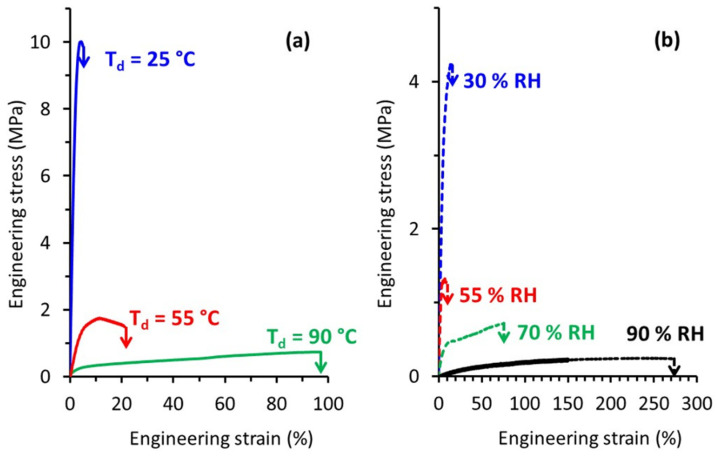
Engineering stress-strain curves of TPS samples stretched (**a**) at different T_d_ and RH = 70% (**b**) at different relative humidity and T_d_ = 80 °C.

The mechanical behavior of TPS was investigated in this study over a wide range of both temperature and relative humidity. Figure 4a presents typical engineering stress-strain curves recorded during tensile tests carried out at different draw temperature (T_d_) while the relative humidity was kept constant at 70% RH. At T_d_ = 25 °C, TPS exhibited a brittle behavior in agreement with previously published results. The increase of T_d_ to 55 °C led to a significant decrease of the applied stress, as well as to an increase of the deformation at break. A plastic deformation regime with uniform deformation could be evidenced under these drawing conditions. Finally, a further increase of T_d_ to 90 °C involved a huge increase in the deformation at break beyond 90%. At this T_d_, TPS behaved as a rubbery material, as revealed by homogeneous deformation at almost constant and low stress. The evolution of the mechanical behavior of TPS with temperature was similar to the one observed for other commodity plastics, with a brittle to ductile transition, which could be ascribed to the glassy to rubbery transition as temperature increased. 

The influence of relative humidity on the tensile behavior was also investigated. Typical engineering stress-strain curves recorded at T_d_ = 80 °C for relative air humidity ranging from 30% RH to 90% RH are shown in Figure 4b.

Basically, the evolution of the mechanical response was similar to the one observed as a function of temperature. At low RH, TPS behaved like a brittle material, while for higher RH we were dealing with a rubbery material. A deformation at break as high as 270% could be reached when TPS was drawn at a relative humidity of 90%. Worth noting is that even if high deformation ratios were reached there was no indication of any strain-hardening process as is generally observed in the case of other initially amorphous polymers stretched above T_g_ [28]. This strain-hardening phenomenon generally occurs at the end of drawing and originates from a strong orientation of the macromolecular network towards the draw axis and/or from a strain-induced crystallization process. 

Table 1 summarizes the values of Young modulus E, tensile strength σ_break_ and strain at break ε_break_ of TPS as a function of temperature and relative humidity. The average values of these mechanical characteristics are in the same order of magnitude as those reported in literature for glassy and rubbery TPS [13,19]. 

Figure 4 clearly shows that both drawing temperature and relative humidity at which tensile tests were carried out played a key role on the observed mechanical behavior. Since temperature governs the molecular mobility of the polymer, increase of T_d_ involved an increase in molecular mobility. In the meantime, the relative humidity would directly impact the water content of TPS and consequently its T_g_. In order to quantify the influence of the water content on T_g_, we determined the glass transition temperature of the TPS samples as a function of the stretching conditions, using the procedure described in the experimental part. The resulting data are gathered in Table 2.

**Figure 5:**
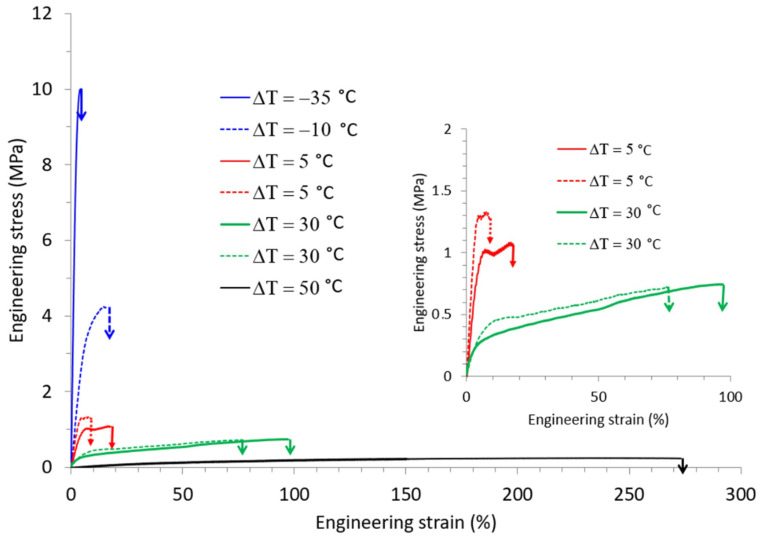
Engineering stress-strain curves of TPS samples stretched at different ΔT, the gap between the drawing temperature T_d_ and the glass transition temperature T_g_.

Depending on the temperature and humidity conditions in the stretching oven, the water content and, thus. T_g_ clearly varied. This consequently showed that not only T_d_ but also the relative humidity had to be considered in order to account for the mechanical behavior of TPS. In order to probe the interplay between these two parameters, special attention was paid to the influence of ΔT, that is the difference between T_d_ and T_g_ on the mechanical response. Miri et al. reported in a previous work that ΔT was a key parameter to rationalize the plastic deformation behavior of PA6, another water sensitive polymer [29]. In order to check whether the same conclusions might be drawn in the case of TPS, the tensile data of Figure 4a,b were combined and expressed in terms of ΔT, as displayed in Figure 5.

It appears that the results were quite representative of the generally observed mechanical behavior of amorphous polymers. For negative ΔT (i.e., stretching in the glassy state) the material was brittle and was characterized by high applied stresses and low strains at break. By contrast, for positive ΔT (i.e., for stretching in the rubbery state), the behavior was rubbery like and increasing ΔT involved an increase in the strain at break. Quite strikingly, the stress-strain curves obtained for the same ΔT were superimposed; the example for ΔT = 30 °C convincingly illustrates the fact that under very different stretching conditions the mechanical response was the same. ΔT appeared, therefore, as the key parameter to rationalize the mechanical behavior of TPS under complex environmental conditions (T, RH). 

### 3.3. Structural Evolution during Stretching

**Figure 6:**
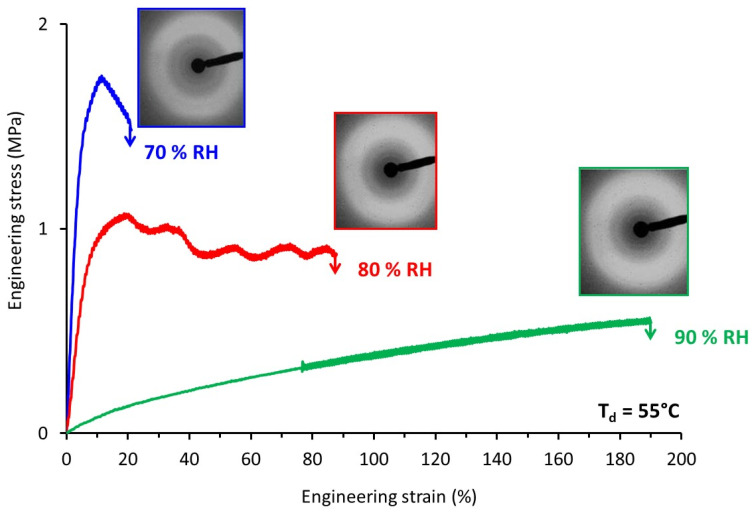
Engineering stress-strain curves of TPS samples stretched at T_d_ = 55 °C and relative air humidity varying from 70 to 90% and corresponding WAXS patterns recorded on the post-stretched samples (the draw axis is horizontal).

In order to best-understand the tensile response, the structural evolution upon stretching of TPS was followed by means of WAXS experiments. TPS samples stretched at T_d_ = 55 °C under different relative air humidity were analyzed by means of ex-situ WAXS. Figure 6 depicts the engineering stress-strain curves of the samples and the associated WAXS patterns of the drawn samples. The drawing conditions tested allowed the covering of a wide range of mechanical behavior, from brittle to significantly ductile. The WAXS patterns recorded looked quite similar whatever the stretching conditions. Analyses of the azimuthal intensity profiles (not shown here) confirmed the absence of significant molecular orientation, or of any signs of the formation of an ordered phase. This supported the fact that no strain-hardening was observed on the stress-strain curves. 

## 4. Conclusions

This work was devoted to the study of the influence of the stretching conditions on the mechanical behavior of TPS. Besides the characterization of the viscoelastic properties of the material as a function of its water content we showed that water molecules do not simply act as a common plasticizer, by reducing T_g_, but also strongly modify the macromolecular network by changing the intermolecular H-bonding density. This results in a decrease of the storage modulus in the rubbery plateau with the addition of the water content. Regarding the uniaxial tensile behavior, it was also clearly evidenced in this work that both the drawing temperature and relative humidity at which TPS is drawn infers on the mechanical behavior. It was shown that the effect of these two parameters can be summarized by considering the difference between the drawing temperature and the glass transition temperature. It is the key parameter to rationalize the mechanical behavior of TPS. The structural characterization of stretched samples shows that even if large plastic deformation ratios can be achieved there are no significant signs of macromolecular orientation upon stretching. 

## Figures and Tables

**Table 1:** Influence of T and RH on Young modulus E, tensile strength σ_break_ and strain at break ε_break_.

Stretching Conditions	R.H. = 70%			T = 80 °C			
	T (°C)			R. H. (%)			
	25	55	90	30	55	70	90
E (MPa)	420 ± 100	30 ± 10	6 ± 1	80 ± 10	45 ± 10	7 ± 3	0.4 ± 0.1
σ_break_ (MPa)	10 ± 1	1.5 ± 0.5	0.8 ± 0.3	4 ± 1	1.3 ± 0.5	0.7 ± 0.3	0.3 ± 0.2
ε_break_ (%)	4 ± 1	22 ± 6	100 ± 10	15 ± 4	12 ± 6	80 ± 25	240 ± 80

**Table 2:** Influence of T and RH on water uptake and glass transition temperature.

T_d_ (°C)	R.H. (%)	Water Content (g H_2_O/g Total) (% ± 1)	T_g_ (°C ± 5)	ΔT = T_d_ − T_g_ (°C)
25	70	16	60	−35
55	70	17	50	5
90	70	16	60	30
80	30	12	90	−10
80	55	14	75	5
80	70	17	50	30
80	90	20	30	50

## Data Availability

The data is this study are available on reasonable request from the corresponding authors.

## References

[1] Souza R.C.R., Andrade C.T. (2002). Investigation of the gelatinization and extrusion processes of corn starch. Adv. Polym. Tech..

[2] Stepto R.F.T. (2003). The processing of starch as a thermoplastic. Macromol. Symp..

[3] Stepto R.F.T. (2006). Understanding the processing of thermoplastic starch. Macromol. Symp..

[4] Stepto R.F.T. (2009). Thermoplastic starch. Macromol. Symp..

[5] Van Der Burgt M.C., Van Der Woude M.E., Janssen L.P.B.M. (1996). The influence of plasticizer on extruded thermoplastic starch. J. Vinyl Addit. Technol..

[6] Bendaoud A., Chalamet Y. (2013). Effects of relative humidity and ionic liquids on the water content and glass transition of plasticized starch. Carbohydr. Polym..

[7] Sankri A., Arhaliass A., Dez I., Gaumont A.C., Grohens Y., Lourdin D., Pillin I., Rolland-Sabaté A., Leroy E. (2010). Thermoplastic starch plasticized by an ionic liquid. Carbohydr. Polym..

[8] Wang H., Huang M. (2007). Preparation, characterization and performances of biodegradable thermoplastic starch. Polym. Adv. Technol..

[9] Forssell P.M., Hulleman S.H.D., Myllärinen A.P.J., Moates G.K., Parker R. (1999). Ageing of rubbery thermoplastic barley and oat starches. Carbohydr. Polym..

[10] Gaudin S., Lourdin D., Forssell P.M., Colonna P. (2000). Antiplasticisation and oxygen permeability of starch-sorbitol films. Carbohydr. Polym..

[11] Mikus P., Alix S., Soulestin J., Lacrampe M.F., Krawczak P., Coqueret X., Dole P. (2014). Deformation mechanisms of plasticized starch materials. Carbohydr. Polym..

[12] Thunwall M., Boldizar A., Rigdahl M. (2006). Compression molding and tensile properties of thermoplastic potato starch materials. Biomacromolecules.

[13] Van Soest J.J.G., De Wit D., Vliegenthart J.F.G. (1996). Mechanical properties of thermoplastic waxy maize starch. J. Appl. Polym. Sci..

[14] Hulleman S.H.D., Janssen F.H.P., Feil H. (1998). The role of water during plasticization of native starches. Polymer.

[15] Lourdin D., Valle G.D., Colonna P. (1995). Influence of amylose content on starch films and foams. Carbohydr. Polym..

[16] Dieulot J., Skurtys O. (2013). Classification, modeling and prediction of the mechanical behavior of starch-based films. J. Food Eng..

[17] Véchambre C., Buléon A., Chaunier L., Jamme F., Lourdin D. (2010). Macromolecular orientation in glassy starch materials that exhibit shape memory behavior. Macromolecules.

[18] Véchambre C., Buléon A., Chaunier L., Gauthier C., Lourdin D. (2011). Understanding the mechanisms involved in shape memory starch: Macromolecular orientation, stress recovery and molecular mobility. Macromolecules.

[19] Coativy G., Gautier N., Pontoire B., Buléon A., Lourdin D., Leroy E. (2015). Shape memory starch-clay bionanocomposites. Carbohydr. Polym..

[20] Bizot H., Le Bail P., Leroux B., Davy J., Roger P., Buleon A. (1997). Calorimetric evaluation of the glass transition in hydrated, linear and branched polyanhydroglucose compounds. Carbohydr. Polym..

[21] Lourdin D., Coignard L., Bizot H., Colonna P. (1997). Influence of equilibrium relative humidity and plasticizer concentration on the water content and glass transition of starch materials. Polymer.

[22] Chung H.J., Lim S.T. (2006). Physical aging of amorphous starches (a review). Starch/Staerke.

[23] Ferry J. (1980). Viscoelastic Properties of Polymers.

[24] Semsarzadeh M.A., Mehrabzadeh M., Arabshabi S.S. (2002). Dynamic mechanical behavior of the dioctyl phthalate plasticized polyvinyl chloride-epoxidized soya bean oil. Eur. Polym. J..

[25] Sheth M., Kumar R.A., Davé V., Gross R.A., McCarthy S.P. (1997). Biodegradable polymer blends of poly (lactic acid) and poly (ethylene glycol). J. Appl. Polym. Sci..

[26] Graessley W.W., Edwards S.F. (1981). Entanglement interactions in polymers and the chain contour concentration. Polymer.

[27] Bayer R.K., Cagiao M.E., Baltá Calleja F.J. (2006). Structure development in amorphous starch as revealed by X-ray scattering: Influence of the network structure and water content. J. Appl. Polym. Sci..

[28] Stoclet G., Seguela R., Lefebvre J.M., Elkoun S., Vanmansart C. (2010). Strain-induced molecular ordering in polylactide upon uniaxial stretching. Macromolecules.

[29] Miri V., Persyn O., Lefebvre J.-M., Seguela R. (2009). Effect of water absorption on the plastic deformation behavior of nylon 6. Eur. Polym. J..

